# Molecular Markers in Oral Lichen Planus - Insight into Pathogenesis

**DOI:** 10.1007/s12105-025-01775-1

**Published:** 2025-03-26

**Authors:** Maria Zaharieva Mutafchieva, Milena Nenkova Draganova, Georgi Tomchev Tomov

**Affiliations:** 1 Department of Periodontology and Oral Mucosa Diseases, Faculty of Dental Medicine, Medical University of Plovdiv, Plovdiv, 4000 Bulgaria; 2 Department of Medical Biology, Faculty of Medicine, Medical University of Plovdiv, Plovdiv, 4000 Bulgaria; 3 Research Institute, Medical University of Plovdiv, Plovdiv, 4000 Bulgaria; 4 Department of Healthcare and Social Work, New Bulgarian University, Sofia, Bulgaria

**Keywords:** Oral lichen planus, Apoptosis, p53, p63, bcl-2, Ki-67, COX-2

## Abstract

**Purpose:**

Oral lichen planus (OLP) is a chronic inflammatory disease, characterized by immune-mediated basal keratinocyte apoptosis. In recent years the importance of programmed cell death for the tissue destruction in OLP has been disputed, while at the same time an increased proliferative index has been reported in the epithelium of these lesions. OLP is considered as a precancerous condition. This study investigated the expression of pro-apoptotic, anti-apoptotic and proliferative markers in OLP lesions in an attempt to understand more about the pathogenesis and malignant potential of the disease.

**Methods:**

Twenty patients with histologically confirmed OLP were compared to ten healthy controls through immunohistochemical analysis of the levels of p53, p63, bcl-2, Ki-67 and COX-2.

**Results:**

The results demonstrated significantly decreased expression of p63 in OLP lesions compared to normal oral mucosa. The levels of p53, bcl-2, Ki-67, and COX-2 were not significantly different from those in the control group. A significant association was found between p63 and Ki-67 (*p* = 0.001), as well as between p63 and p53 (*p* = 0.016). Expression of the inflammatory COX-2 and the apoptotic p53 appeared to be independent of each other (*p* = 0.44). The intensity of expression of any of the five analyzed markers was not related to the severity of the clinical manifestation.

**Conclusions:**

The obtained results suggest that apoptosis may not be the dominant mechanism in the disease’s pathogenesis. Decreased expression of p63 on the other hand appears to play an important role. Among the possible effects of this protein deficiency are activation of programmed cell death, cell cycle arrest, cellular senescence, or anoikis; suppression of cell proliferation or changes in cell differentiation. The observed reduction in p63, Ki67 and bcl-2 levels predisposes to epithelial thinning, erosions and/or ulcers. For the presented OLP cohort, there was no molecular evidence of increased malignant potential of the lesions.

## Introduction

Oral lichen planus (OLP) is a chronic-inflammatory disease of unknown etiology. Oral lesions may occur in isolation or along with skin lesions [1]. The total global prevalence of this disease is 1.01%, but it seems to demonstrate geographic predominance, being most common in Europe [1]. Middle-aged women are usually affected [2]. Clinically, OLP presents in six different forms, which can be grouped into (hyper)keratotic (reticular, papular, plaque-like) and atrophic-erosive types (atrophic, bullous and erosive forms) [1, 3]. “Wickham striae”, representing white keratotic lines in a lace-like pattern are considered a hallmark of the disease and are usually found in all clinical forms - alone or at the periphery of atrophic, erosive and bullous lesions [3]. Symptoms can range from none or a mild burning sensation to severe pain that interferes with speech and eating. Some lesions may be recalcitrant to treatment.

The overall consensus regarding pathogenesis is that OLP is an immune-mediated disease, initiated by the expression of an unknown antigen on the surface of the basal keratinocytes of the oral epithelium. A number of viruses (HSV, HPV etc.), microbial antigens and commensal fungus as Candida albicans have been listed as putative exogenous triggers [4]. Additionally, altered endogenous self-peptide or a heat shock protein may also be presented on the keratinocytes to instigate an immune reaction [4, 5]. In addition to antigen presentation, the stimulated keratinocytes release a plethora of pro-inflammatory cytokines and chemokines, activating a cascade of molecular events, that lead to the recruitment of T cells to the OLP lesion sites, which are then activated by antigen binding to major histocompatibility complex (MHC)-1 on keratinocytes. In the final stage of the immune response CD8^+^ T-cells destroy basal keratinocytes through activation of cell death program (apoptosis). Civatte bodies, representing apoptotic epithelial cells, are considered a hallmark in OLP. Therefore, it is widely accepted that keratinocyte apoptosis is a significant aspect in the pathogenesis of OLP [4–6].

Apoptosis is a highly regulated form of cell death. Unlike necrosis, apoptosis is a natural physiologic process routinely carried out in multicellular organisms and normally does not elicit inflammatory response [7]. Programmed cell death can be induced by DNA damage, hypoxia, uncontrolled cell proliferation, toxins, stress, hormones, growth factors, radiation etc., which determines the importance of the process in cell protection and cellular genetic errors elimination [8]. There are two major apoptosis pathways: extrinsic and intrinsic. The intrinsic pathway, also known as non-receptor-mediated pathway, involves series of events, leading to increased permeability of the mitochondrial transmembrane, which is associated with the release of pro-apoptotic proteins into the cytosol. The presence of Cytochrome C in the cytosol binds Apaf-1 and caspase 9, which in turn activates the effector caspase-3 [7, 8]. The extrinsic pathway is accomplished through an interaction of death ligands (Fas ligands, TNF ligands) with death receptors (Fas receptor, TNF receptors). This allows activation of the initiator caspase 8, which in turn activates caspase 3, responsible for the final execution (7, 8). Caspases (initiator caspases − 8, -9, -2, -10, -11 and executioner caspases − 3, -6, -7) are essential for the implementation of apoptosis [7].

Both the intrinsic and extrinsic pathways are regulated by a class of proteins, that can be categorized into pro-apoptotic and anti-apoptotic. BCL-2 family of proteins are pivotal in terms of the regulation of apoptosis by the activities of their pro-apoptotic (Bax, Bak, Bcl-10, Bad etc.) and anti-apoptotic (Bcl-2, Bcl-x, Bcl-xl, Bf-1etc.) members. In addition, the tumor suppressor p53 has been recognized for its critical function to initiate apoptosis. Furthermore, there are many other cell cycle regulators that can affect apoptosis (e.g. p21, p63, COX-2 etc.) [7].

Cells undergoing apoptosis exhibit some morphological changes, including cell blebbing, shrinkage, pyknosis, nuclear fragmentation and formation of small vesicles known as apoptotic bodies [7]. In the course of cellular self-destruction, six phases are distinguished, on which the principles for diagnosing the apoptotic process are based: (1) triggering stimulus (2) signal transduction phase (FasL/FasR or CD40L/CD40 expression); (3) effector phase involving the caspase family of proteins (Caspase 3 expression); (4) DNA degradation and fragmentation (TUNEL test, Agarose gel electrophoresis); (5) formation of apoptotic bodies (quantification of Civatte bodies in histological specimens) and (6) efferocytosis - the removal of the apoptotic cells by phagocytes.

Defects in cell death program may lead to broad spectrum of diseases. In this regard, uncontrolled cell division with evasion of apoptosis are hallmarks of cancer. Excessive apoptosis, on the other hand, is also a pathological condition [7]. Abnormalities in the apoptotic process have been blamed for the onset of autoimmune diseases, as dwindled apoptosis of immune cells will ensure the immortality of the latter [7]. In addition, excessive apoptosis with inefficient ingestion of the dying cells by phagocytes may lead to necrosis and autoantigens, released from dying cells activate T- and B cells and induce autoimmunity [7].

Keratinocytes apoptosis is the main consequence of the immune aggression seen in OLP [2, 4]. It is generally accepted that CD8 + T-cells induce apoptosis in the basal keratinocytes by several mechanisms: (1) secretion by T cells of TNF-α, which binds TNF-α receptor 1 (TNFR-1) on the keratinocyte surface; (2) interaction of CD8 + T-cell-expressed Fas ligand (FASL) with FAS receptor (FasR) on the keratinocyte surface; (3) binding of CD40 and CD40L expressed by keratinocytes and cytotoxic T-cells, respectively; (4) T-cell-secreted Granzyme-B entering the keratinocyte cytoplasm through perforin-induced membrane pores [4–6, 9]. Perforin–granzyme-mediated apoptosis is exclusively employed in cytotoxic killing mediated by T cells and occur through mechanisms different from those of the extrinsic and intrinsic pathways, described above [7]. In addition, T cell-independent mechanisms for inducing apoptosis have been described in OLP. For instance, the disruption of the basement membrane by mast cell proteases (MMP-9) may also trigger cell death [2, 4, 5]. Furthermore, decreased expression of adhesion molecules, responsible for cell-cell and cell to basement membrane contacts may also induce keratinocyte apoptosis via anoikis in OLP lesions [4, 10].

Despite the great scientific progress on the topic, the precise molecular mechanisms driving programmed cell death in OLP are still elusive. None of the studied apoptotic proteins (p53, p63, p21, bcl-2, BAX etc.) has demonstrated consistent expression finding, so that to be utilized as diagnostic or prognostic marker of the disease. Moreover, in recent years the thesis of pathologically enhanced apoptosis being present in OLP has been disputed. The rationale: failure to demonstrate high levels of caspase 3 (an end protein in the apoptotic process) in all OLP patients [11], weak TUNEL test positivity [11], as well as increased expression of proliferative markers (PCNA, Ki-67), reported by some authors [12–16]. Additionally, if most of the basal keratinocytes die by apoptosis, the regenerative compartment of the epithelium would be lost, which ultimately would lead to atrophy or ulcerations [13]. While on the contrary, epithelial thickening with hyperkeratosis, parakeratosis and acanthosis are prominent pathоhistological features of OLP lesions.

Finally, OLP is included in the group of oral potentially malignant disorders (OPMDs) [17]. However, the malignant potential of the disease is a subject of great controversy. Proteins, that control apoptosis, cell growth, cell proliferation and differentiation also play a crucial role in oncogenesis. Increased expression of p53 [18, 19], p63 [20], bcl-2 [21], COX-2 [22, 23] and Ki-67 [24] has been found in OSCC and thus these proteins are considered as possible prognostic markers indicating malignant transformation of OPMD.

The aim of the study was to determine the importance of the processes apoptosis and cell proliferation in the pathogenesis of OLP.

## Materials and Methods

### Study Design

20 patients with symptoms and oral manifestation corresponding to the clinical diagnosis of OLP were included in the study. The participants were divided into six groups according to the clinical form of the disease: patients with reticular -, papular -, plaque-like-, atrophic-, erosive- or bullous form. If concomitant lesions of different types were observed in the mouth, the patient was categorized according to the most severe one. Biopsies of sufficient size were taken for histological confirmation of the diagnosis and immunohistochemical analysis. We initially examined the expression of epidermal factor p63 (results discussed in another paper of ours) [25], anti-apoptotic bcl-2 and proliferative Ki67 [26]. For a more thorough analysis of the apoptotic process, we later decided to further evaluate the immune reactivity for two other leading markers - p53 and COX-2 in the same patients and looked for correlations between all five. For this purpose, additional tissue sections were obtained retrospectively from the stored paraffin blocks. All the samples were collected with the informed consent of the patients. To compare the levels of these five biomarkers (p53, p63, bcl-2, Ki-67, COX-2) in OLP lesions with those in normal oral mucosa (NOM), biopsies were also taken from ten healthy volunteers.

The study was conducted in accordance with the Declaration of Helsinki. The research protocol was approved by the Ethics Committee of Medical University Plovdiv (R3716/07.10.2014).

### Research Focused Questions

- Could any of the included proteins (p53, p63, bcl-2, Ki-67, COX-2) be used as a distinctive diagnostic or prognostic biomarker for OLP patients.
- Can immunohistochemical examination of p53, p63, bcl-2, Ki-67, COX-2 levels help to understand the pathogenesis of the disease - pathologically enhanced apoptosis or increased proliferation rate is the leading process.
- Is there molecular evidence for increased malignant potential of these OLP lesions.

### Research Contingent

- Patients group − 20 patients with oral lichen planus.
- Control group − 10 subjects with normal oral mucosa.

An informed written consent was obtained from all participants in the study.

### Inclusion Criteria

- Patients with all clinical forms of OLP - reticular, papular, plaque-like, atrophic, bullous or erosive forms.
- Patients with histologically confirmed diagnosis OLP, based on the modified WHO criteria of Van der Meij [27].
- Healthy volunteers who needed any surgical procedure associated with tissue excision (mainly tooth extraction). Only tissues with no visible signs of inflammation were collected.

### Exclusion Criteria

- If oral lichenoid reactions were suspected - lesions in close proximity to amalgam fillings оr a temporal relationship between the introduction of some drug and the onset of the disease [28].
- Clinically and histologically ambiguous cases of OLP, suspected of being other mucosal diseases (such as pemphigus, pemphigoid, lupus erythematosus) and requiring further investigation by immunofluorescence technique, etc [28].
- If signs of dysplasia were observed in the histological specimens.
- History of taking corticosteroids, non-steroidal anti-inflammatory drugs or other immunosuppressants in the last 6 months.

### Incisional Biopsy with ER: YAG Laser

Biopsy of 3 mm diameter and 1.5 mm depth was taken using Er: YAG laser at the following parameters: pulse mode, 35 Hz, 7 W, 200 mJ. To facilitate histological diagnosis, the pathognomonic Wickham striae were included in all tissue samples, while areas of deep ulcerations were avoided. The biopsy was stored in 10% formalin at neutral pH (6.8–7.2) (biopsy: solution ratio 1:10) until embedded in paraffin.

### Immunohistochemical Examination

4 μm tissue sections were applied to special poly-L-lysine coated slides. The latter were then deparaffinized in xylene and rehydrated with successively decreasing percentages of ethanol (100%, 96%, 90%, 80%, 70%). Antigen retrieval was performed in 0.01 M citrate buffer (pH 6.0) using a water bath for 45 min at 95 °C. Endogenous peroxidase was blocked using 3% hydrogen peroxide for 5 min, followed by protein block (Bio SB-Mouse/rabbit polydetector HRP/DAB kit (Cat.N: BSB 0201 S). Incubation with the primary antibodies was performed at room temperature in a humid chamber for 45 min. Then, the samples were incubated with biotinylated secondary antibody for one hour and with streptavidin-HRP for 30 min at room temperature (RT). Detection of the antigen-antibody reaction was carried out by 3, 3’-diaminobenzidine (DAB). Cell nuclei were counterstained with hematoxylin.

A Nicon eclipse Ni-U light microscope was used. The presence of a brown staining, was considered a positive reaction. For selecting of a representative field the following sequence was used: 1- under a small (X10) magnification, the area with the most intense staining was selected; 2 - under magnification X40, the percentage of positive cells was determined using a semiquantitative scale: (-) negative result - < 5% stained cells; (+) weak expression − 5–25% positive cells; (++) moderate expression − 25–50% positive cells; (+++) strong expression - staining in > 50% of the cells.

### Statistical Analysis

Statistical analysis was performed using SPSS 11.5 Inc., Chicago, IL, USA, Excel 7.0 VB for applications, and GraphPad Prism 3.0 (GraphPad Soft, San Diego, CA, USA). Differences between cases and controls, as well as between the clinical forms were analyzed by a Chi-square test (X^2^). Associations among immunohistochemical parameters were established by Spearman correlation test. P values less than 0.05 were considered statistically significant.

## Results

The OLP contingent consisted of 20 patients, 17 females and 3 males, with the following age distribution: >61 (7 patients); 51–60 (3 patients); 41–50 (7 patients); 31–40 (2 patients); <30 (1 patient). 6 patients were diagnosed with reticular form, 1 - with papular form, 2 - with plaque like form, 5 - with atrophic form, 5 - with erosive form and 1 with bullous form of OLP. The control group consisted of 10 age (≥ 18-year-old; mean age 54.9) and sex (8 women:2 men) matched healthy volunteers.

A comparison of the expression intensity of all five markers in NOM versus OLP lesions is shown in Table 1. Statistically significant difference was found only in the levels of the epidermal factor p63.

**Table 1:** Comparison of p53, p63, bcl-2, Ki-67 and COX-2 expression between healthy controls and OLP patients

Variable	*Healthy controls* (*n* = 10)		*OLP patients* (*n* = 20)		*P* value
	*n*	%	*n*	%	
Expression of p53 - + ++ +++	6 1 0 3	60 10 0 30	11 1 5 3	55 5 25 15	*p* = 0.32
Expression of p63 - + ++ +++	0 0 0 10	0 0 0 100	7 1 2 10	35 5 10 50	*p* < 0.05
Expression of bcl-2 - + ++ +++	3 2 4 1	30 20 40 10	14 3 3 0	70 15 15 0	*p* = 0.12
Expression of Ki-67 - + ++ +++	0 1 5 4	0 10 50 40	2 5 4 9	10 25 20 45	*p* = 0.28
Expression of COX-2 - + ++ +++	5 2 2 1	50 20 20 10	5 8 3 4	25 40 15 20	*p* = 0.47

Expression of p53, p63, bcl-2, Ki-67 and COX-2 in healthy controls (n=10) and patients with OLP (n=20)

**Fig. 1:**
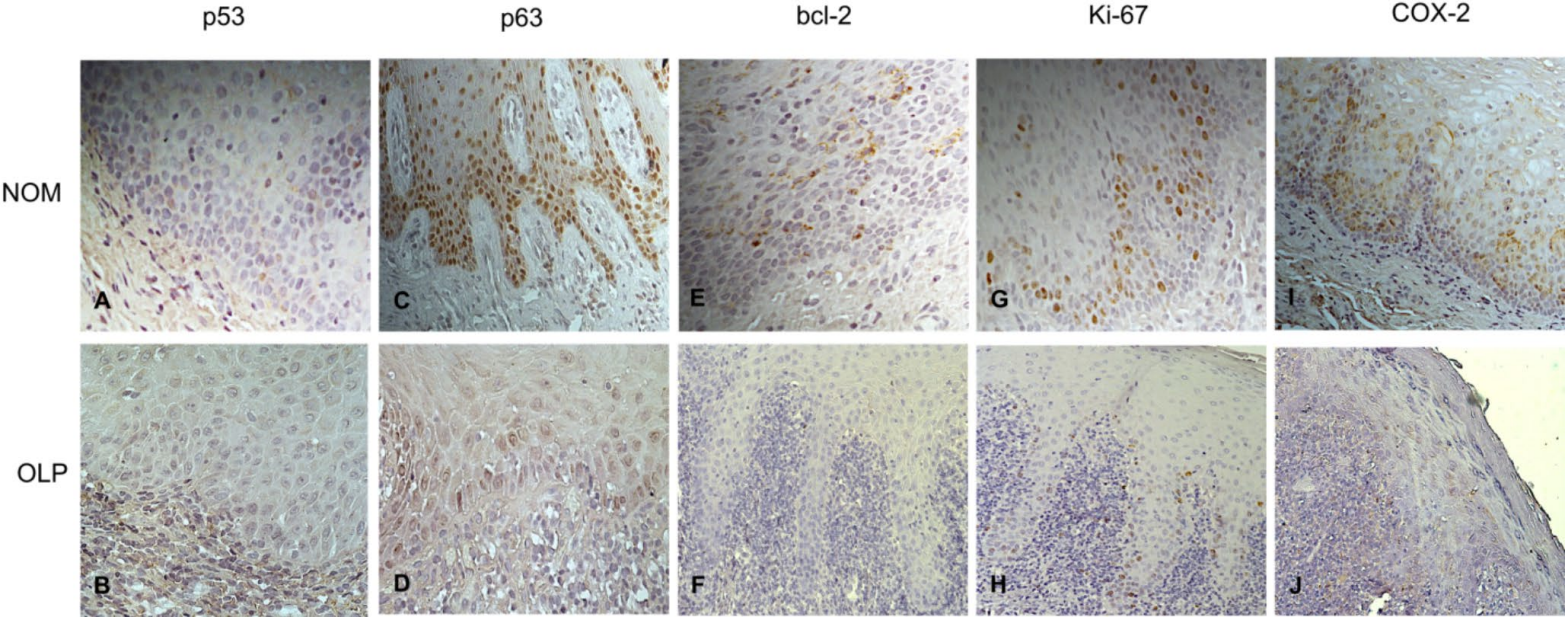
Immunohistochemical staining for the molecular markers in normal oral mucosa (NOM) and oral lichen planus: (**A**): weak expression (+) of p53 in healthy epithelium; (**B**): lack of p53 positivity (-) in OLP lesion; (**C**): p63 staining in more than 50% of the keratinocytes (+++) in NOM; (**D**): decreased levels of p63 (++) in OLP tissues; (**E**): expression of bcl-2 (++) in healthy epithelium; (**F**): Absence of bcl-2 immunoreactivity (-) in OLP specimen; (**G**): Strong expression of Ki-67 in healthy controls; (**H**): decrease in the intensity of reaction (++) for this proliferative marker in OLP; (**I**): High levels of COX-2 (+++) in NOM; (**J**): Weak expression of COX-2 in the epithelium of OLP lesion

Strong expression (+++) of p63 was observed in all samples (100%) from normal oral mucosa. A significant reduction of the marker was revealed in the epithelium of OLP lesions (*p* < 0.05), as 35% of the specimens were p63 negative (-) and another 15% demonstrated weak or moderate expression (Fig. 1).

No statistically significant difference in p53 expression was found between patients with OLP and healthy controls (*p* = 0.32). In more than half of the tissue sections in both groups, the marker was absent (Fig. 1). In the cases with positive p53 reaction, the percentage of stained cells was comparable between the groups.

Bcl-2 was expressed in 70% of the specimens from normal oral mucosa, with varying degrees of intensity. Contrariwise, 70% of the OLP samples were bcl-2 negative (Fig. 1). There was a distinct (although not statistically significant) reduction in protein levels in the mucosa of patients with OLP (*p* = 0.12).

Moderate to strong Ki-67 staining was observed in all healthy controls. The applied Chi-square test revealed no significant difference in Ki-67 expression between the patients and control groups (р=0.28). However, there were cases (35%) with mild or absent Ki-67 immune reaction among the OLP contingent (Fig. 1).

Surprisingly, the inflammatory marker COX-2 was expressed in 50% of healthy tissue sections. In the OLP group a positive immune reaction was detected in 75% of the cases, with weak (+) expression being more frequent (40%) (Fig. 1). The staining intensity in normal oral mucosa and in OLP lesions was comparable (р=0,47).

**Fig. 2:**
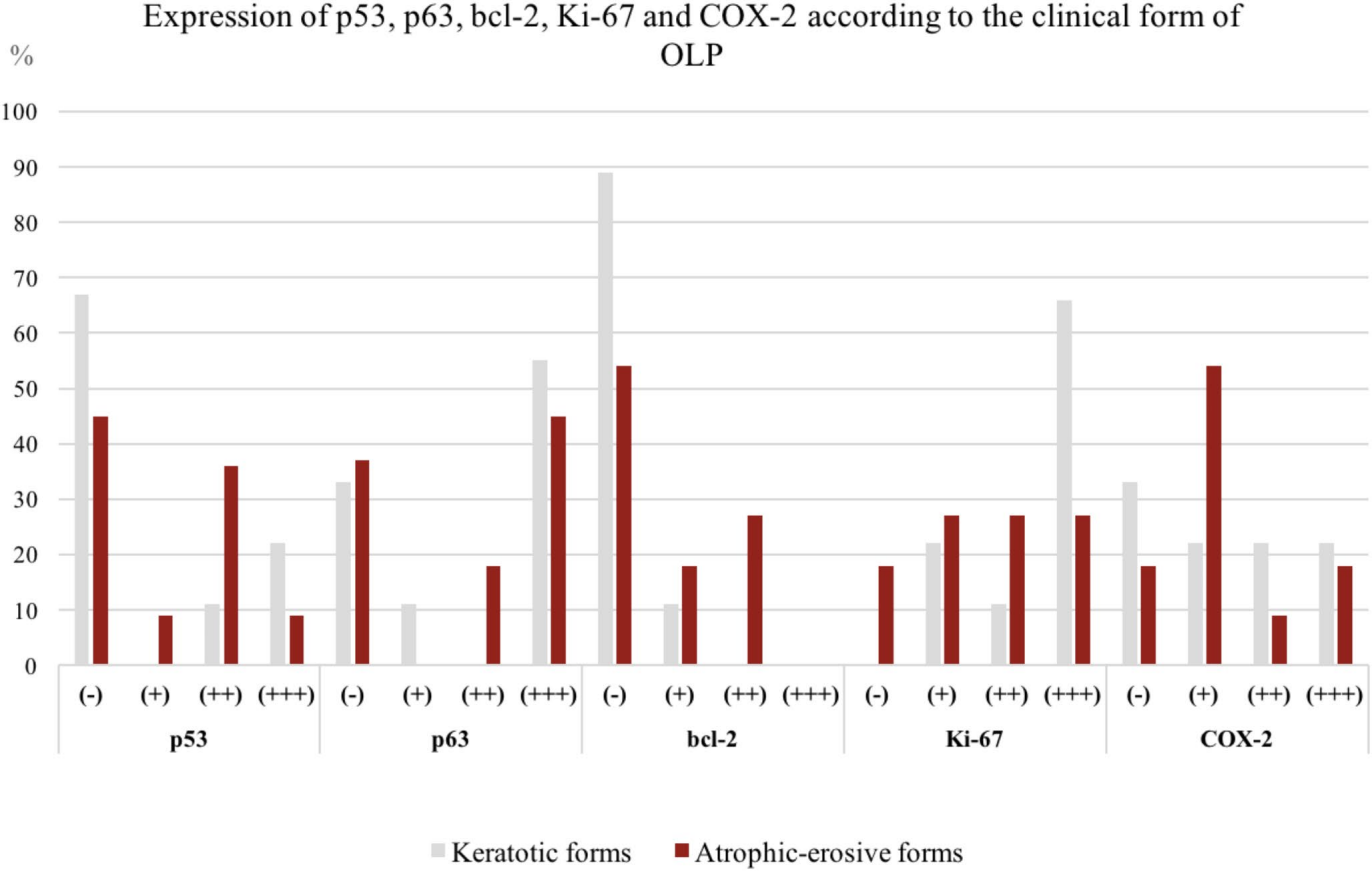
Expression of p53, p63, bcl-2, Ki-67 and COX-2 in keratotic and atrophic-erosive forms of OLP

None of the five markers analyzed was associated with the severity of the clinical manifestation, as statistical analysis revealed no differences in the levels of p53, p63, bcl-2, Ki-67 and COX-2 between keratotic and atrophic-erosive forms of OLP (Fig. 2).

**Fig. 3:**
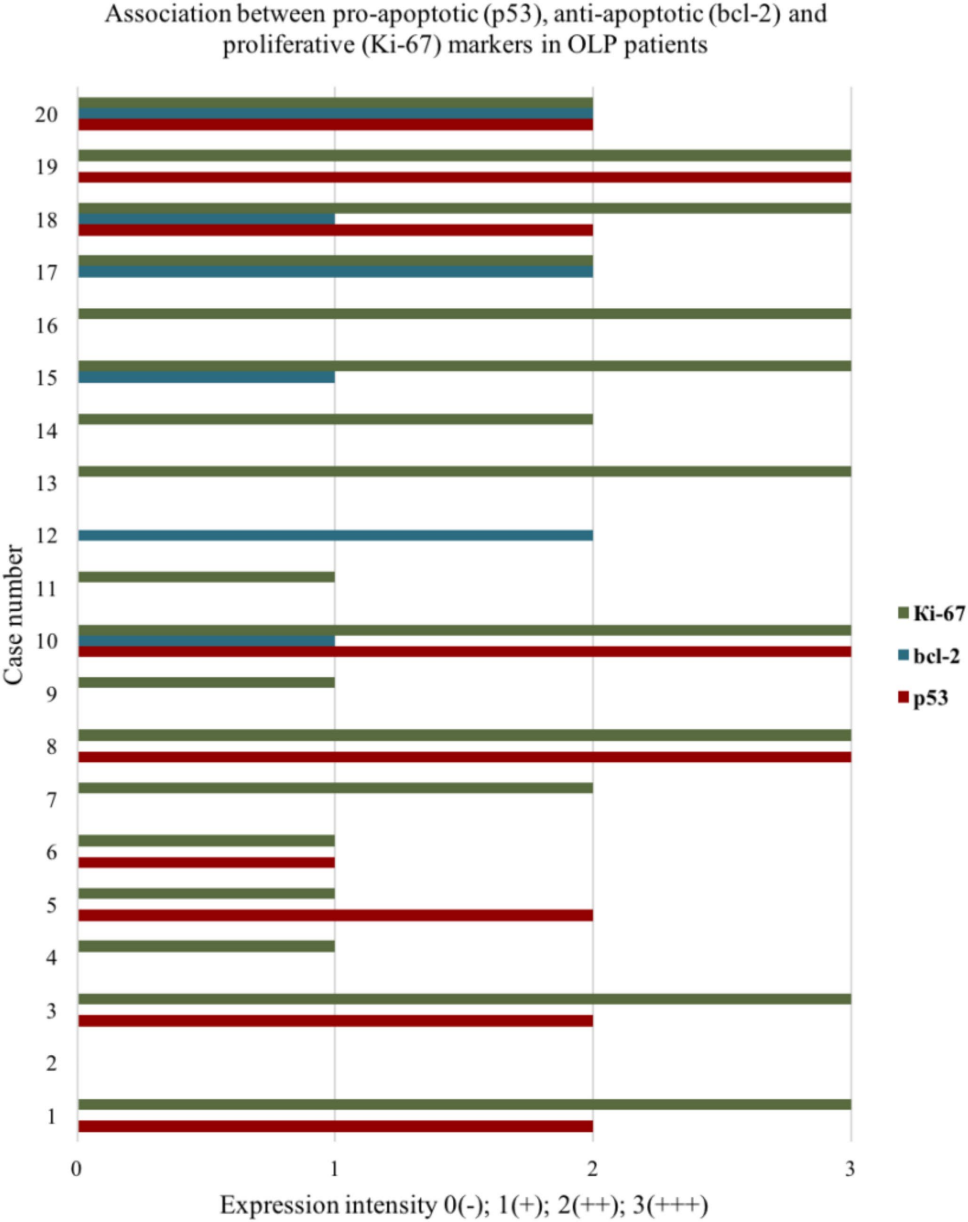
Association between pro-apoptotic (p53), anti-apoptotic (bcl-2) and proliferative (Ki-67) markers for each of the 20 patients with OLP

Figure 3 visualizes the relationship between pro-apoptotic, anti-apoptotic and proliferative markers for each of the 20 patients with OLP included in the study. Evidence in favor of an apoptotic process (increased expression of p53 with a reduced proliferative index and absence of anti-apoptotic molecules) were present in only two of the cases (No. 5 and 6).

The applied Spearman correlation test demonstrated a significant association between p63 and Ki-67 (*p* = 0.001), as well as between p63 and p53 (*p* = 0.016). Expression of the inflammatory COX-2 and the apoptotic p53 appeared to be independent of each other (*p* = 0.44).

## Discussion

Pathologically enhanced apoptosis in the epithelium of OLP lesions has been reported in the literature [4, 29]. In this regard, p53-mediated apoptotic pathway is among the most studied mechanisms in the pathogenesis of the disease. The “Guardian of the genome” p53 plays a crucial role in cell cycle control, triggering either cell cycle arrest or apoptosis, thereby preventing the replication of damaged DNA. During the cell cycle arrest this marker induces transcription of proteins involved in DNA repair [18]. However, if the DNA damage is irreversible, p53 activates programmed cell death through several mechanisms: precedes the release of cytochrome C and activation of procaspase-3; suppresses anti-apoptotic Bcl‐2 and Bcl‐xL and up-regulates pro-apoptotic BAX [30]. The reported p53 immunopositivity in OLP lesions varies from 18 to 100% among different studies [31]. According to Basheer S et al. a possible explanation for this large variation in the data in the literature may be the inclusion of oral lichenoid reaction (OLR) and oral lichenoid dysplasia (OLD) in the OLP contingent due to misdiagnosis; as well as different p53 antibodies and antigen retrieval methods used [31]. Another issue is whether given levels of p53 should be considered as pathologically increased expression. The confusion stems from the lack of constant expression pattern for the marker in normal oral mucosa. For example, Hadzi-Mihailovic M et al. demonstrated that 80% of OLP specimens were p53-positive but did not interpret these results as significantly increased expression [18]. In contrast, in the study of Basheer S. et al. immunoreactivity for p53 was found in 40% of the OLP cases and was considered overexpression [31]. The rationale: in the mentioned studies, p53 was expressed in 53.85% [18] and in none [31] of the control sections of normal oral mucosa, respectively. The prevailing view is that in normal cells p53 staining is absent [31, 32] or weak [19, 33, 34] and thus any protein levels other than these in OLP are considered pathologically enhanced [19, 32, 34]. p53 overexpression in OLP, on the other hand, has different explanations given by the scientific literature. Gudkov AV et al. stated that the inflammatory infiltrate seen in OLP causes keratinocyte DNA damage and higher levels of p53 represent a protective response to maintain genomic stability by activating cycle arrest or programmed cell death [35]. Another concept is that p53 overexpression is a physiologic reaction to the reported hyperproliferative state in OLP and the goal is again DNA damage repair or elimination [16, 18]. However, most authors interpret the higher detection of p53 in OLP tissues as an indication of а neoplastic transformation of these lesions [18, 30, 31]. Overexpression of p53 has been reported not only in OSCC [18] but also in dysplastic lesions [19], suggesting that such an immunohistochemical finding may correspond to an early stage of oral cancerization of OLP lesions. Furthermore, according to Hadzi-Mihailovic M et al., the observed p53 in OLP samples represents mainly a mutated form of this protein [18]. Mutations or other inactivation mechanisms prevent the p53 system from working properly, allowing abnormal cells to proliferate, which results in cancer [18, 36, 37]. In this regard, Calenic B et al. demonstrated increased p53 levels with low expression of caspase-3 (the final executor enzyme of apoptotic pathways) and concluded that p53 apoptotic system was activated but did not execute [11]. These facts suggest that the p53 network as a barrier to tumor initiation may not function well in patients with OLP.

In the present study p53 was expressed in 45% of the OLP samples with staining intensity as follows: 15%(+++); 25%(++); 5%(+). These protein levels are significantly lower compared to p53 positivity rate reported by other authors − 69.9% Shailaja G [34]; 71.4% Leyva-Huerta E [32]; 80% Hadzi-Mihailovic M [18] and 100% Safadi R [19], but are in line with those of de Sousa F et al. (41.67%) [38] and Basheer S. et al. (40%) [31]. However, the applied Chi-square test in the present study showed no significant difference in either the percentage of p53 positive samples or the staining intensity between the OLP group and healthy controls (Table 1). The explanation is that we detected p53 immunoreactivity in 40% of cases with normal oral mucosa, of which 30% (*n* = 3) demonstrated strong intensity (+++). According to these results the expression of the pro-apoptotic p53 is not pathologically altered in OLP lesions.

As already mentioned above, p53 is not the only protein that mediates the apoptotic process. In this regard, reduced expression of the anti-apoptotic bcl-2 is an indicator for a high degree of apoptosis [30]. The detailed mechanism by which Bcl‐2 inhibits apoptosis is still uncertain, but it was suggested that this protein blocks mitochondrial cytochrome C translocation and simultaneously prevent caspase activation [30]. Therefore, the absence of this anti-apoptotic marker is associated with a loss of its anti-cell death function, which in turn promotes apoptosis. Data in the literature regarding bcl-2 expression in the epithelium of OLP lesions vary widely [13, 32, 39–41] - from significantly increased [30] to significantly decreased [6] compared to that in normal oral mucosa. In the present study, we found a marked (although not statistically significant) reduction in bcl-2 levels in the keratinocytes of OLP tissues compared with those in healthy epithelium (Table 1). This finding is consistent with the results of other studies [13, 29, 32, 40] and may predispose to the occurrence of apoptosis.

Activation of the programmed cell death is a protective mechanism to avoid proliferation of cells containing abnormal DNA. Therefore, if apoptosis occurs the proliferation index should be low. In support of this thesis, Pérez-Sayáns M et al. reported that the proliferative marker Ki-67 was positively correlated with the anti-apoptotic bcl-2 and negatively with the pro-apoptotic BAX [6]. However, a great number of studies have shown increased proliferation in OLP than in normal oral mucosa [13, 15, 16, 34, 42]. Ki-67 is crucial in the proliferation process. Its expression begins in the G1 phase, increases gradually in the subsequent–S and G2 phases, and reaches its maximum during cell division (M phase) [15]. According to the results of this study, Ki-67 levels did not differ significantly between the working (OLP contingent) and control groups (Table 1). Moreover, the expression intensity for the marker was slightly lower in OLP lesions.

General limitation of most of the prior studies, in our opinion, is that conclusions are made based on the immunohistochemical results total for the studied contingent, while the molecular effects depend on precise interplay between the different markers which is not the same among all patients. To determine the significance of the processes apoptosis and cell proliferation in the pathogenesis of the disease, we examined the relationship between p53, bcl-2 and Ki-67 for each of the twenty patients with OLP (Fig. 3). Considering all above a combination of elevated p53, absence of bcl-2 and low Ki-67 levels points in favor of an activated cell death program.

In the specimens of four of the OLP patients (n-7,13,14,16) a positive reaction was found only for Ki-67 with moderate (++) to strong (+++) intensity; in another two (n-15,17) this Ki-67 expression was also accompanied by staining for the anti-apoptotic bcl-2. In the absence of pro-apoptotic p53, we concluded for all of them that there was an intense process of epithelial renewal. Absence (-) or weak (+) expression of proliferative Ki-67, combined with absence of bcl-2 expression was found in cases n- 2, 4, 9, 11. However, in these cases there was also no p53 reaction, therefore it cannot be claimed that the reduced proliferative index is due to pathologically increased apoptosis. The pro-apoptotic p53 was expressed in nine of the twenty OLP tissue sections. However, in seven of the p53 positive cases there was an intense immunoreaction for Ki-67 (n-1, 3, 8, 10, 18, 19, 20) and additionally in three of them (n-10, 18, 20) bcl-2 was also expressed. This combination of proteins did not correspond to an enhanced apoptosis. Therefore, it can be speculated that for these cases p53 had a preventive function against the amplification of the genetic errors that have occurred, but did not lead to cell death. Only in two of the analyzed cases (n-5 and 6) the combination of markers pointed in favor of activated mechanisms of programmed cell death– there was mild to moderate expression of p53, accompanied by low Ki-67 levels and absence of bcl-2. Clinically, these patients demonstrated erosive and atrophic lesions.

From the above analysis, it can be concluded that for the presented sample of patients there was no evidence of an activated p53-dependent apoptosis pathway. This is not the first study to not associate apoptosis with p53 positivity. Hadzi-Mihailovic M et al. reported low percentage and weak intensity of p53-positive cells in OLP specimens with highly expressed Civatte bodies (CB) [18]. Since p53 is a pro-apoptotic protein, and CB are symbols of apoptosis, positive correlation between these parameters should be expected. Therefore, it could be assumed that apoptosis may occur, but other molecules may be implemented in its activation. In this regard, p63 is also an apoptotic marker [11]. In the present study we found significantly lower levels of p63 in the epithelium of OLP lesions compared to those in normal oral mucosa (Table 1). The established deficiency of this ectodermal factor in OLP seems to be an important molecular mechanism in the pathogenesis of the disease, as none of the other four markers analyzed demonstrated a significant expression change compared to the control group. Furthermore, data in the literature are relatively consistent regarding lower levels of the protein in this patients [10, 33]. A few sources indicate increased expression [11]. P63 is a transcription factor of P53 family, which determines lots of overlapping functions with p53. p63 is required for both proliferation and differentiation of the keratinocytes. It is also involved in cell cycle arrest, apoptosis and cell senescence and maintains epithelial integrity by regulating the expression of different adhesion markers. The mechanisms for triggering p63-dependent programmed cell death are not fully understood. It is considered that p63 is required for the epidermal apoptosis mediated by p53 [11]. Ebrahimi M et al. stated that a combination of low p63- and high p53 levels is needed to induce apoptosis [33]. The applied Spearmаn correlation test in the present study demonstrated a significant association between these two proteins in OLP lesions (р<0.02). However, p63 correlated positively with p53, as seven out of nine p53-positive cases highly expressed also p63(+++). Calenic B. et al. reported a p63/p53 correlation with strong immunoreactivity for both markers in patients with OLP, which was however accompanied by low levels of caspase 3 [11]. Therefore, with similarly high expression of p53 and p63, apoptosis does not occur. On the other hand, an in vitro study demonstrated that simultaneous blocking of both proteins did not result in cell death but, on the contrary, partially corrected the epithelial hypoplasia caused by p63 deficiency [43]. It could be concluded that in the implementation of the molecular mechanisms of the disease, p53 and p63 act in a coordinated manner, but do not lead to cell death.

There are also less known mechanisms of p63-mediated apoptosis. Anoikis is a form of programmed cell death that occurs in anchorage-dependent cells when they lose contacts to their neighboring cells or extracellular matrix [20]. Decreased expression of adhesion proteins such as E-cadherin and b-catenin has been reported in OLP [10]. E-cadherin and b-catenin are p63-dependent adhesion molecules [10]. The reduced expression of p63, found in the present study, indirectly points to a lack of adhesion proteins. Therefore, it could be speculated that the observed tissue destruction in OLP is precisely due to anoikis.

Cyclooxygenase 2 (COX-2) is also involved in the process of apoptosis. COX-2 is an enzyme responsible for the synthesis of bioactive prostanoids from arachidonic acid and is implicated in the processes of inflammation, reproduction, and carcinogenesis. Lichen planus is a chronic inflammatory disease and predictably, COX-2 levels have been reported to be higher in these patients [41, 44]. Overexpression of COX-2 may represent a marker for inhibition of apoptosis [9]. Among the mechanisms by which this inflammatory marker blocks programmed cell death are: reduction of cytochrome C and caspase 3 activity and induction of increased expression of anti-apoptotic bcl-2 [45]. In the conducted study we found increased levels of COX-2, which however, did not differ significantly from those in normal oral mucosa (Table 1). Furthermore, no significant correlation was demonstrated between this inflammatory marker and pro-apoptotic p53 (*p* = 0.44). Additionally, in the present study bcl-2 levels were low, so it cannot be argued that COX-2 enhanced its expression. Therefore, there is no evidence to support COX-2 promoted cell survival.

In summary, for the presented OLP cohort, the immunohistochemical analysis, including five apoptotic markers, did not confirm the significance of programmed cell death in the pathogenesis of the disease. In making this conclusion, we still keep in mind that the expression pattern of pro- and anti-apoptotic proteins conditionally indicates whether apoptosis has occurred or not. In order to correctly determine the extent of apoptosis, additional diagnostic techniques are required, such as examination of caspase 3 expression; DNA fragmentation (TUNEL method); chromatin condensation (ssDNA); number and distribution of apoptotic bodies (Civatte bodies). Bascones C. et al. applied a TUNEL assay and Caspase-3 expression examination in their study and, like us, concluded that the apoptotic phenomenon was of little quantitative importance in OLP [46]. Instead, they reported basal expression of p21. The latter is a cyclin dependent kinase inhibitor, that arrests the cell cycle in G1, inducing DNA repair or senescence. Low rate of apoptosis with increased expression of p21 [30] have been demonstrated also in other studies [13]. p21 is known to be negatively regulated by p63 [43]. In this regard, the p63 deficiency found in the present study may indirectly lead to cell cycle arrest through the resulting intense expression of p21. Therefore, we share the position of Bascones C. et al. that epithelial cells under attack in OLP respond more frequently with arrest or senescence than with apoptosis [46].

The results of our study did not support the notion of pathologically increased proliferation in OLP, as Ki-67 expression in these lesions was even lower (although not statistically significant) compared to that in normal oral mucosa. In vitro studies have shown that lack of p63 resulted in severe epithelial hypoplasia [43]. We also found a significant positive correlation between p63 and Ki-67 expression. Therefore, the established reduced expression of p63 may result in suppression of cell proliferation, which predisposes to epithelial thinning.

Keratotic forms of OLP are characterized by thickening of the epithelium. Thus, increased expression of proliferative markers and decreased expression of apoptotic proteins can be expected. The opposite expression pattern is more likely to be detected in the more severe - atrophic-erosive forms of the disease. However, statistical analysis showed no significant difference in p53, p63, bcl-2, Ki-67, and COX-2 levels between these two groups, thus none of them can be used as prognostic marker for disease severity.

As stated above, increased expression levels of p53, p63, bcl-2, COX-2 and Ki-67 are considered indicative of dysplastic transformation of the tissue. Since the expression of p53, bcl-2, COX-2, and Ki-67 in the studied OLP lesions was not significantly different from that in normal oral mucosa and the levels of the epidermal factor p63 were found to be even lower in these patients we can conclude that for the study cohort presented, there was no evidence of carcinogenesis.

The main drawbacks of the studies focusing on immunohistochemistry in OLP (including the present one) are: (1) There are no generally accepted values ​​for the expression intensity of a given marker in normal oral mucosa. This leads to large discrepancies in the interpretation of the results in OLP: same values ​​are interpreted as overexpression by some authors and as downexpression by others; (2) Ideally, control biopsies and those taken from patients should be from the same anatomical area, whereas in most studies the control samples are from gingiva adjacent to the teeth planned for extraction, which is firstly a keratinized mucosa and secondly may show signs of low-grade inflammation; (3) The antibodies used to detect the markers cannot distinguish between the wild-type and mutated form of p53, nor between the TAp63 and ΔNp63 isoforms of p63, which have different, often opposite, effects. Further studies addressing the aforementioned issues are needed to improve the accuracy of immunohistochemical analysis as a diagnostic tool.

## Conclusion

No pathognomonic immunohistochemical finding was found in OLP lesions, as none of the five markers analyzed demonstrated consistent expression pattern. The intensity of expression of p53, p63, bcl-2, Ki-67 and COX-2 was not associated with the severity of the clinical manifestation and thus had no prognostic character. There was no evidence of an activated p53-dependent apoptosis pathway. Expression of this marker more likely signifies activated defense mechanisms (such as cell cycle arrest and cellular senescence), aimed at correcting DNA defects. Decreased expression of p63 in the epithelium of OLP patients appears to be an important molecular mechanism in the pathogenesis of the disease and may lead to apoptosis, cell cycle arrest, cell senescence, anoikis, suppression of cell proliferation or alterations in cell differentiation. The observed combination of simultaneous reduction in p63, Ki67 and bcl-2 levels predisposes to epithelial thinning, erosions and/or ulcers. The obtained results of the study did not support the assumption of an increased malignant potential of these lesions.

Studies with increased sample size and applying additional specific diagnostic techniques for apoptosis detection (TUNEL method; caspase 3 expression etc.) are needed to confirm the reliability of the results obtained in the present study.

## Data Availability

The data that support the finding of this study are available from the corresponding author, [MM], upon reasonable request.

## Code Availability.

No software application was used in the preparation of this article.
